# The Olive Oil Phenolic *S*-(-)-Oleocanthal Suppresses Colorectal Cancer Progression and Recurrence by Modulating SMYD2-EZH2 and c-MET Activation

**DOI:** 10.3390/nu17030397

**Published:** 2025-01-22

**Authors:** Md Towhidul Islam Tarun, Heba E. Elsayed, Hassan Y. Ebrahim, Khalid A. El Sayed

**Affiliations:** School of Basic Pharmaceutical and Toxicological Sciences, College of Pharmacy, University of Louisiana at Monroe, 1800 Bienville Drive, Monroe, LA 71201, USA; tarunmt@warhawks.ulm.edu (M.T.I.T.); hebassan_2005@yahoo.com (H.E.E.); hebrahim@ulm.vcom.edu (H.Y.E.)

**Keywords:** colorectal cancer, c-MET, extra-virgin olive oil, EZH2, *S*-(-)-oleocanthal, recurrence, SMYD2

## Abstract

**Background/Objectives**: Colorectal cancer (CRC) is the third most common cancer in the US and the second leading cancer-associated mortality cause. Available CRC therapies achieve modest outcomes and fail to prevent its recurrence. Epidemiological studies indicated that the Mediterranean diet rich in olive oil reduced CRC incidence. This study aimed at the identification and assessment of active anti-CRC olive phenolics. **Methods**: The MTT, wound-healing and colony formation assays were used to discover and assess the in vitro anti-CRC activity of olive phenolics. A nude mouse xenografting model was used to assess the in vivo CRC progression and recurrence suppressive activity of OC in pure and crude forms. OC was isolated from olive oil using liquid–liquid extractions. **Results**: Screening of olive phenolics for in vitro antiproliferative activity against a diverse panel of CRC cell lines identified the extra-virgin olive oil (EVOO) *S*-(-)-oleocanthal (OC) as the most active hit. OC showed IC_50_ values of 4.2, 9.8, 14.5, and 4.9 μM against HCT-116, COLO-320DM, WiDr, and SW48 CRC cells, respectively. The lysine methyltransferases SMYD2 and EZH2, along with the receptor tyrosine kinase c-MET proved aberrantly dysregulated in invasive and metastatic CRC. SMYD2 and c-MET were validated as OC molecular targets in multiple malignancies. Daily oral 10 mg/kg OC treatments over 15 days suppressed 72.5% of the *KRAS* mutant HCT-116-Luc cells tumors weight in male nude mice. Continued OC daily oral use after primary tumor surgical excision over an additional 40 days significantly suppressed the HCT-116-Luc locoregional tumor recurrence and totally prevented the distant tumor recurrence. The SMYD2-EZH2 expressions and c-MET activation were notably suppressed by OC treatments in vitro and in collected animal primary tumors. **Conclusions**: OC and olive phenolics are potential nutraceutical interventions useful for CRC control and the prevention of its relapse.

## 1. Introduction

Colorectal cancer (CRC) is the third most common cancer in the United States and the second leading cancer-associated mortality cause in both genders, with 154,270 estimated new cases in 2025, along with 28,900 and 24,000 deaths among males and females, respectively [1,2,3,4]. CRC five-year disease-free survival rates are 91–18.9% for stages I–IV, respectively [5,6]. The <55 year-old cases are progressively increasing in the US and worldwide [1,6,7,8]. CRC has a high recurrence rate and growing resistance to existing therapies, leading to treatment failure. Detected in 40% of CRC, *KRAS* (Kirsten rat sarcoma viral oncogene homolog) mutations in codons 12 or 13 induce constitutive activation, inducing RAS/RAF/MAPK pathways, causing worse prognosis and high resistance to EGFR-targeted therapies [9]. Current therapies do not effectively eradicate CRC, and therefore novel interventions are direly needed.

Cellular lysine methylation is driven by the protein lysine methyl-transferases (PKMTs) like the SET and MYND-containing protein (SMYD) enzyme family. SMYD PKMTs are critical for gene regulation, chromatin remodeling, transcription, signal transduction, cell cycle control, and DNA damage response [10,11,12,13,14]. SMYD2 methylates H3K36 and H3K4, in the presence of HSP90, histone and non-histone proteins like the enhancer of zeste homolog 2 (EZH2), p53, and others [12]. The master transcriptional regulator histone PKMT EZH2 promotes histone H3 K27 trimethylation [12]. SMYD2 directly methylates EZH2 at K307, promoting tumorigenesis [12,15,16]. Lysine methylation by SMYD2-EZH2 modifies protein substrate bulkiness, hydrophobicity, and molecular recognition by methyl-lysine readers [10,16]. The SMYD2 gene in 1q32-q41, a chromosomal region, is amplified in CRC and other cancers [10,11,12,13,14,15,16]. c-MET is a transmembrane receptor tyrosine kinase that can be activated by the hepatocyte growth factor (HGF) [17,18,19,20]. HGF aberrant expression by CRC activates c-MET, triggering downstream PI3K/AKT and RAS/RAF/MAPK pathways promoting tumor growth, survival, motility and angiogenesis [17,18,19,20]. Clinically, SMYD2 and c-MET are aberrantly dysregulated in patients with invasive CRC, and proved to be correlated with poor patient prognosis [21,22].

Epidemiological studies suggested that the Mediterranean diet reduced the incidence of CRC and other cancers [23,24]. Extra-virgin olive oil (EVOO) is a key Mediterranean diet ingredient [23,24]. *S*-(-)-Oleocanthal (OC) is a monophenolic secoiridoid naturally occurring in EVOO, with documented anticancer activities [25]. OC inhibited MIP-1α, IL-6, 5-lipoxygenase, HSP90, mTOR, STAT3, ERK1/2, and AKT in different cancer types [25]. OC was identified as a competitive c-MET kinase domain inhibitor, and therefore showed potent suppressive effects on the breast cancers progression and recurrence [26,27,28]. Daily 10 mg/kg oral OC treatments inhibited the metastatic castration-resistant prostate cancer progression and recurrence via direct SMYD2 inhibition, and suppressed its expression level [29]. EVOO was proven in vitro to be active against multiple CRC cell lines [30]. OC showed moderate in vitro cytotoxicity against the CRC cells HT29 and SW480 through the generation of reactive oxygen species [31]. OC suppressed the in vitro proliferation of Caco-2 and HT-29 CRC cell lines with EC_50_ of 34.3 and 26.3 µM, respectively [32]. OC, oleuropein, and hydroxytyrosol were proposed as the most anti-CRC olive phenolics, with diverse molecular targets [33]. This study screened olive phenolic anti-CRC activities, and identified OC as the most active hit. Thus, the study comprehensively validated the pure OC and OC-rich EVOO-derived polyphenol rich fraction (PPRF) as orally effective anti-CRC progression and recurrence leads by targeting SMYD2-EZH2 expression and c-MET activation.

## 2. Materials and Methods

### 2.1. Chemicals and Reagents

Most reagents and chemicals were acquired from VWR International (Suwanee, GA, USA) unless otherwise indicated. *S*-(-)-oleocanthal (OC) and PPRF were extracted from the Greek EVOO (The Governor, Corfu, Greece) using the liquid–liquid extraction methodology described earlier by this study team, followed by SP70 resin entrapment and elution of PPRF by acetone after water displacement [34]. OC was finally purified on Sephadex LH20, in a ratio of 1 g PPRF-100 Sephadex LH20 and elution with CH_2_Cl_2_ with increasing amounts of EtOAc. The established OC purity of ≥99% was validated by the q^1^H NMR analysis detailed earlier [34]. The quantitation was essentially based on the integration ratio of the OC key H-3 aldehydic proton signal at δ 9.23 and the residual CHCl_3_ peak in CDCl_3_ at δ 7.24 [34].

### 2.2. Cell Lines and Culture Conditions

The American Type Culture Collection (ATCC, Manassas, VA, USA) was the commercial source for the non-tumorigenic colon epithelial CCD 841 CoN cells. The human CRC HCT-116 was purchased from Charles River Laboratories, Inc., Wilmington, MA, USA. The human CRC cell lines COLO-320DM, SW48, and WiDr were generously provided by Dr. Yong-yu Liu, School of Basic Pharmaceutical & Toxicological Sciences, College of Pharmacy, University of Louisiana at Monroe, LA, USA. Cells were cultured in Roswell Park Memorial Institute (RPMI-1640) or Dulbecco’s Modified Eagle’s medium (DMEM) with 10% fetal bovine serum (FBS) supplement, along with penicillin G (100 U/mL), and 100 ng/mL streptomycin. Cells were maintained at 37 °C, with 5% CO_2_ in a humidified incubator. Cells were washed with Ca^2+^ and Mg^2+^-free phosphate-buffered saline (PBS) and incubated in 0.05% trypsin possessing 0.02% ethylenediaminetetraacetic acid (EDTA) at 37 °C for 3–5 min before subsequent subculture.

### 2.3. Experimental Treatments

A stock of 25 mM OC was prepared by dissolving OC in sterile dimethyl sulfoxide (DMSO). This stock solution was utilized to prepare diverse treatment concentrations for various experiments. The final concentration of DMSO was maintained at the same level in all treatment groups within each experiment, and was not allowed to exceed 0.1% in the treatment media.

### 2.4. Cell Viability Assay

About 1 × 10^4^ cells of each cell line/well, six replicates per experimental group, were seeded into 96-well plates in 10% FBS RPMI-1-640 media and incubated overnight to recover and attach. Cells were then divided into different treatment groups and exposed to respective control or experimental treatments the next day, with various OC or vehicle control concentrations for 24 or 48 h in media. 3-(4,5-dimethylthiazolyl2)-2,5-diphenyltetrazolium bromide (MTT) was applied to quantify the viable cell number at the end of the experiment [28,29,33]. A final concentration of 1.0 mg/mL MTT was added to each well. After incubation for 4 h at 37 °C, the media was then removed, and 100 µL of DMSO was added to each well to solubilize the formazan crystals. Optical density determined was at 570 nm on a microplate reader (BioTek, Winooski, VT, USA) for quantification, using an established standard calibration curve [28,29,34].

### 2.5. Wound-Healing Assay

The CRC cells HCT-116 were plated in 24-well sterile flat-bottom plates (3 replicates/treatment group). Cells were left overnight to form subconfluent monolayers in each well. Wounds were then inflected in each cell monolayer by a sterile pipette tip (200 µL). The media was then removed and the cells washed twice with sterile PBS. The cells were incubated in 0.5% serum-containing culture media, with added various OC treatments. Cells were left incubated for 24 h or until the vehicle control (VC) wells’ wound closed. The media was then removed and the cells washed with precooled sterile PBS, fixed with absolute ethanol, and then stained with Giemsa. The healing of each wound was visualized at 0 and 24 h, or until the full closure of the control wound, using a Nikon Ti2-A Inverted Intelligent microscope (Nikon Instruments Inc., Melville, NY, USA). Each wound was digitally imaged and healing distance was estimated by comparing the wound width after 24 h, or at the end of the experiment, with the width of each wound at the beginning of the treatment (zero time). The acquired % migration was determined by setting the t0 gap width as 100%. Reproducibility was assured by repeating each experiment in triplicate.

### 2.6. Colony Formation Assay

The KRAS-mutant CRC cells HCT-116 were seeded in 12-well plates (1000 cells/well). Cells were treated after 24 h with either PPRF (µg/mL) or pure OC (µM) at various concentrations. The media was replaced every 72 h with or without treatments. Each experiment continued until the no-treatment control showed distinct colony formation, mostly within 12–14 days. Crystal violet was used to stain each of the treatment colonies, which were then photographed. The number of colonies was scored using CFU Scope quantification software [29]. Results were reported as the number of colony-forming cells per well in percentages, and normalized to the no-treatment control representing 100%.

### 2.7. Western Blot Analysis

About 1 × 10^6^ HCT-116 cells were plated in 10 cm culture plates in RPMI 1640 containing 10% FBS, and cells were left overnight to attach. The cells were washed with PBS and treated with vehicle control or various PPRF or OC concentrations for 48 h. The cells were then collected and washed with sterile cold PBS, twice. The cells were resuspended and lysed at 4 °C for 30 min in radioimmunoprecipitation assay (RIPA) buffer (Qiagen Sciences Inc., Valencia, CA, USA). Cell lysates were centrifuged for 10 min at 14,000× *g* and supernatants were stored ultra-frozen at −80 °C. Collected mouse primary tumor samples after surgical excision time were immediately stored at −80 °C until protein extraction. Collected tumors were homogenized in RIPA buffer. Each protein concentration sample was calculated by Pierce BCA Protein Assay (Thermo Fisher Scientific Inc., Rockford, IL, USA). Sample proteins were resolved on 10% sodium dodecyl sulfate-polyacrylamide gel electrophoresis gels, which were then transferred to polyvinylidene difluoride membranes. Membranes were blocked with EveryBlot blocking buffer (Biorad, Hercules, CA, USA) and incubated with each specific primary antibody. Each corresponding horseradish peroxidase-conjugated secondary antibody was used against each primary antibody. The Chemi-Doc XRS chemiluminescent gel imaging system was utilized for sample protein detection and analysis by applying the BioRad Image Lab software v5.2.1 (BioRad, Hercules, CA, USA). β-tubulin visualization was utilized to ensure equal sample loading per individual lane. Each experiment was repeated three times, and representative images were used to present result figures.

### 2.8. Luciferase Labeling of HCT-116 Cells Aided by Lentivirus Transduction

HCT-116 cells were seeded into a 12-well plate till reaching 60–70% confluency. Lentiviral particles carrying luciferase from Kerafast (Boston, MA, USA) were subsequently added to HCT-116 cells. The lentivirus vector was prepared by adding Opti-MEM reduced serum media (1.5 μL/100 μL) and mixed gently on ice. Media subsequently aspirated; the cells were washed with PBS and Opti-MEM media 100 μL, with or without viral particles, and were then added to each well and incubated for 4–6 h. The media was then withdrawn and displaced with complete serum media for a couple of days. Puromycin (Santa Cruz Biotechnology, Dallas, TX, USA) 15 μg/mL was used to select and maintain luciferase-expressing cells. Puromycin-possessing media was frequently displaced, every 2 days. Cellular luciferase activity was assessed by adding 20 μL of 50 mM XenoLight D-luciferin K^+^ salt bioluminescent substrate, PerkinElmer (Waltham, MA, USA), in PBS, into each well, and this was incubated for 6 min at room temperature. The cells were then imaged using a bioluminescence imaging system (PerkinElmer’s IVIS imaging platform) to confirm their bioluminescence tagging [29].

### 2.9. Animal Model and Treatment Mode

Male athymic nude mice (Foxn1^nu^/Foxn1^+^, 4–5 weeks old) were acquired from Envigo (Indianapolis, IN, USA) and received at the University of Louisiana at the Monroe animal facility [27,28,29,34]. The mice were allowed a week for acclimatization before their use in the experimental study. The animals housed in sterile filter-top cages containing autoclaved bedding and provided with sterile water. The vivarium (animal facility) maintained a controlled temperature of 24 ± 2 °C, relative humidity between 50–60%, and a 12 h light–dark cycle. The cages were cleaned and bedding changed twice a week, to minimize animal stress and guarantee best hygiene practice. Animal use included the progression mode after xenografting and the development of palpable tumors and the recurrence mode after primary tumor surgical excisions. All animal experimental procedures were conducted in compliance with the NIH guidelines and approved by the Institutional Animal Care and Use Committee (IACUC) of the University of Louisiana at Monroe, 21-JLY-KES-01. Animal welfare and experimental protocols were followed strictly, to minimize animals’ discomfort and guarantee humane mice use over the study period. Mice clinical health profiles (food and water consumption, defecation, urination, and physical activity) and body weights were carefully monitored, daily, over the study course.

About 2 × 10^6^ HCT-116-Luc cells in 100 µL Matrigel was xenografted subcutaneously into the right back flank of each mouse [27,28,29,34]. Tumor attachment and subsequent progression was monitored daily.

#### 2.9.1. Progression Mode

When each mouse developed a palpable tumor, 50 mm^3^, at the injection site, the mice were randomized into three groups, *n* = 5 each: placebo (dephenolized EVOO) control group, 10 mg/kg OC orally-treated group, and EVOO-derived PPRF standardized for oral dosing at 10 mg/kg OC-treated group. Treatments continued for 15 days.

#### 2.9.2. Recurrence Mode

The mice were anesthetized with isoflurane and subjected to primary tumor surgical excision [28,29,34]. Mice wounds were aseptically clipped. The mice were left to recover under strict clinical monitoring for 24 h post-surgery, for contamination-free adequate wound healing. The next day, each mice group continued with the same oral dosing treatments used in the progression mode for an additional 40 days (placebo control, OC 10 mg/kg and PPRF standardized for OC 10 mg/kg). The animals were then anesthetized with isoflurane and subsequently euthanized. Tumors were surgically excised from each mouse, with dimensions measured every two days using a digital caliper. The tumor volume was deduced based on the following formula: tumor volume (mm^3^) = [(length × width^2^)/2]. The excised tumors and organs were weighed and stored at −80 °C for subsequent total protein extraction and Western blotting analyses [28,29,34].

### 2.10. Statistics

GraphPad Prism software, version 8.4.3. (La Jolla, CA, USA) was used for data analysis. Results are presented as a mean ± standard deviation (SD) for continuous variables. Differences among various treatment and control groups in the in vivo study were calculated by paired Student’s *t*-test, and the *p*-value implications were * *p* < 0.05, ** *p* < 0.01, and *** *p* < 0.001.

## 3. Results

### 3.1. Comparison of the Expression of SYMD2 and c-MET in CRC Cells Versus the Non-Tumorigenic Colon Cells

**Figure 1:**
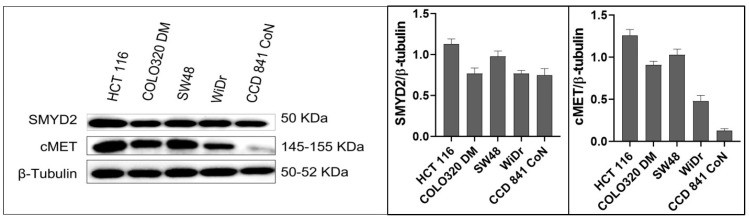
Comparison of SMYD2 and c-MET expressions in CRC cells versus the non-tumorigenic colon epithelial cells CCD 841 CoN, using Western blotting. Bar graphs depict normalized protein levels as mean ± SEM.

Western blots indicated that SMYD2 and c-MET were aberrantly upregulated in CRC cell lines, versus the non-tumorigenic colon epithelial CCD 841 CoN cells (Figure 1). The KRAS*^G13D^*- PIK3CA*^H1047R^* mutated HCT-116 cells [35] with CpG island methylator phenotypes showed nearly 1.5-fold and 5-fold SMYD2 and c-MET expression levels, respectively, versus the non-tumorigenic human colon epithelial CCD 841 CoN cells (Figure 1). The CRC SW48 cells with EGFR mutated and CpG island methylator phenotypes showed the next most dysregulated SMYD2/c-MET expression levels, followed by the neuroendocrine CRC cells double-minute chromosomes COLO-320DM (Figure 1). The BRAF*^V600E^*-PIK3CA*^P449T^*-TP53*^R273H^* mutated WiDr cells [35] showed the least SMYD2 and c-MET expression levels, but this was still much higher than in the CCD 841 CoN cells.

### 3.2. Antiproliferative Activity of Olive Phenolics Against Diverse CRC Cell Lines

**Figure 2:**
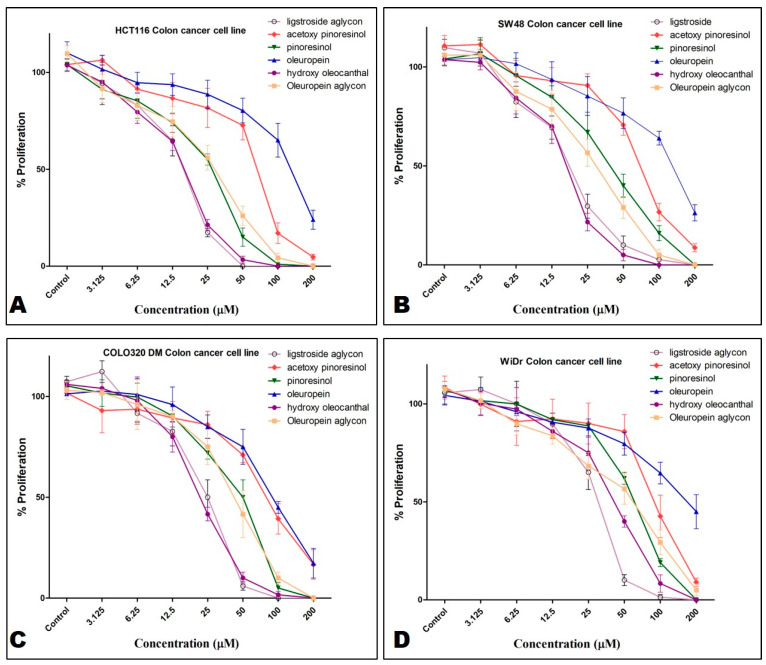
Effect of olive phenolics, excluding OC, against the proliferation of CRC cell lines. (**A**) HCT-116 cells. (**B**) SW48 cells. (**C**) COLO-320DM cells. (**D**) WiDr cells.

**Figure 3:**
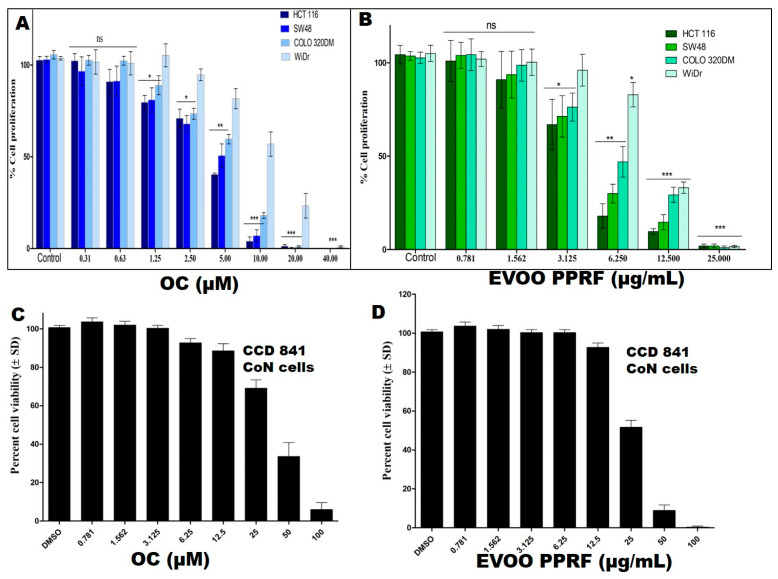
Effects of EVOO phenolics on cell viability. Antiproliferative effects of OC (**A**) and EVOO polyphenol-rich fraction (PPRF) (**B**) against CRC cell lines and the non-tumorigenic colon epithelial cells CCD 841 CoN (**C**,**D**). Statistical significance was assessed using a paired *t*-test: *** *p* < 0.001, ** *p* < 0.01, * *p* < 0.05, and ns indicates no significance.

A small library of olive phenolics was screened against the CRC HCT-116, SW48, COLO-320DM, and WiDr cells at a dose range of 3.125–200 μM, using an MTT assay over a 72 h treatment period (Figure 2). This library included (-)-oleocanthal, (-)-hydroxyoleocanthal, *S*-ligstroside aglycone, *S*-oleuropein aglycon, (-)-oleuropein, (+)-acetoxypinoresinol, and (+)-pinoresinol. Crude polyphenolics rich fraction (PPRF) extracted from EVOO was also tested in parallel, against the same cell lines. OC showed the topmost activity, while oleuropein was the least active (Figure 3A). (-)-Hydroxyoleocanthal and S-ligstroside aglycone were the next most active, but were much less active than OC.

OC revealed IC_50_ values of 4.2, 9.8, 14.5, and 4.9 μM for HCT-116, COLO-320DM, WiDr, and SW48 CRC cells, respectively (Figure 3A). The EVOO PPRF showed IC_50_ values of 3.2, 6.1, 9.8, 3.7, and 11.2 μg/mL for HCT-116, COLO-320DM, WiDr, SW48 CRC cells, and CCD 841 CoN cells, respectively (Figure 3B). Meanwhile, OC and PPRF showed much less effect on the viability of the non-tumorigenic colon epithelial CCD 841 CoN cells with an IC_50_ value of 43.6 μM and 24.8 µg/mL, respectively (Figure 3C,D). This is nearly 7.5-fold the OC IC_50_ value against the HCT-116 CRC cells, proving a notable selectivity towards the malignant cells, rather than the non-tumorigenic cells. The KRAS*^G13D^*-PIK3CA*^H1047R^* mutated HCT-116 cells were the most sensitive, and were therefore selected for subsequent studies.

### 3.3. Effect of OC and PPRF on HCT-116 Migration

The effect of OC and PPRF treatments on the migration of the CRC cell line HCT-116 was assessed by wound-healing assay. OC treatments within a 5–15 μM range and EVOO PPRF in the range of 5–15 μg/mL significantly suppressed HCT-116 CRC cell migration (Supplementary Figure S1).

### 3.4. Effect of OC and PPRF on HCT-116 Cell Colony Formation

The effect of OC and PPRF treatments on the clonogenicity of the CRC cell line HCT-116 was assessed by colony formation assay (Supplementary Figure S2). Subtoxic 2.5 μM OC and 2.5 μg/mL EVOO PPRF treatments significantly suppressed the clonogenicity of the HCT-116 CRC cells, compared to the vehicle control. Higher treatment doses of OC (10 μM) and EVOO PPRF (10 μg/mL) also effectively inhibited HCT-116 colony formation (Supplementary Figure S2).

### 3.5. OC and EVOO PPRF Suppressed the Expression Levels of SMYD2, EZH2, and Activated c-MET in HCT-116 Cells

**Figure 4:**
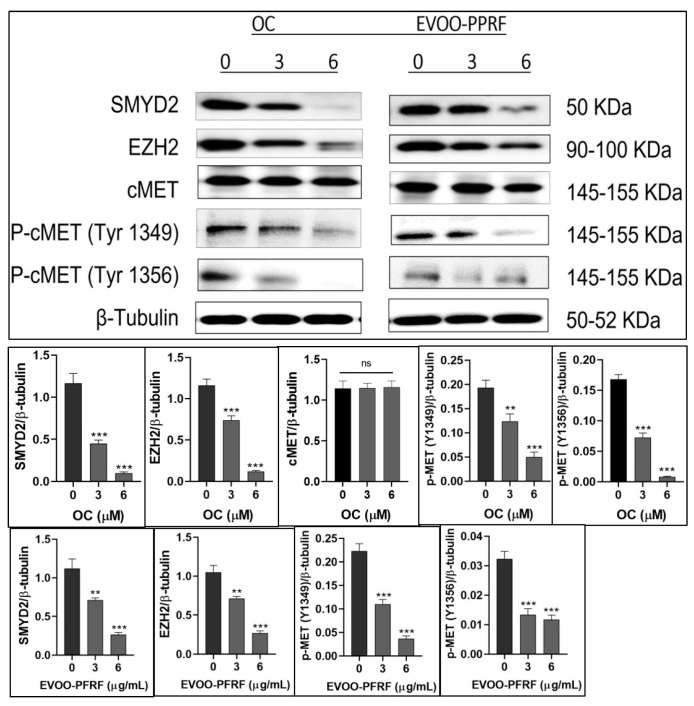
The in vitro OC and PPRF effects on the expression levels of SMYD2, EZH2, total, and activated c-MET in HCT-116 cells. Bar graphs depict normalized protein levels as mean ± SEM. Statistical significance was assessed using a paired *t*-test: *** *p* < 0.001, ** *p* < 0.01, and ns indicates no significance.

Treatment of HCT-116 cells with OC at 3 μM and 6 μM or PPRF at 3 μg/mL and 6 μg/mL effectively suppressed the expression of SMYD2, EZH2, and p-c-MET (Tyrosines 1349 and 1356, Figure 4). These treatments did not affect the expression of total c-MET in HCT-116 cells (Figure 4). OC at 3 μM and 6 μM inhibited 61.1% and 91.1% of total SMYD2 expression levels, respectively (Figure 4). EVOO PPRF at 3 μg/mL and 6 μg/mL treatments suppressed 35.3% and 76.5% of total SMYD2 expression levels, respectively (Figure 4). OC at 3 μM and 6 μM inhibited 34.3% and 90% of EZH2 expression levels, respectively (Figure 4). EVOO PPRF at 3 μg/mL and 6 μg/mL treatments suppressed 34.4% and 73.4% of the EZH2 expression levels in HCT-116 cells, respectively (Figure 4).

OC at 3 μM and 6 μM inhibited 22.9% and 74.3% of activated c-MET-Tyrosine-1349 expression levels, respectively, in HCT-116 cells (Figure 4). Meanwhile, EVOO PPRF at 3 μg/mL and 6 μg/mL treatments suppressed 51.2% and 85.4% of activated c-MET-Tyrosine-1349 expression levels, respectively, in HCT-116 cells (Figure 4). Furthermore, OC at 3 μM and 6 μM inhibited 56.8% and 96% of activated c-MET-Tyrosine-1356 expression levels, respectively, in HCT-116 cells (Figure 4). Meanwhile, EVOO PPRF at 3 μg/mL and 6 μg/mL treatments suppressed 58.3% and 63.9% of the activated c-MET-Tyrosine-1356 expression levels, respectively, in HCT-116 cells (Figure 4).

### 3.6. Comparison of OC Antiproliferative Activity Versus the Chemo and Targeted Therapies

The antiproliferative activity of OC against CRC cells was compared with diverse anticancer drugs, including the microtubule depolymerization disruptor paclitaxel (PTX), doxorubicin (DOX), a DNA base pair intercalator and topoisomerase II inhibitor, osimertinib, a third-generation EGFR inhibitor, 5-fluorouracil (5-FU), a DNA double-strand disruptor and a standard chemotherapy for CRC, SU112274, a class I competitive inhibitor of c-MET kinase domain, and gefitinib, a first-generation EGFR inhibitor (Table 1). OC showed comparable activity compared to 5-FU and the targeted therapies GFT, OSI, and SU11274, but was much less cytotoxic than DOX and PTX (Table 1). The taxane PTX was the most cytotoxic for CRC cells, followed by the anthracycline antibiotic DOX.

### 3.7. Study Design of the In Vivo Assessment of Anti-CRC Activity of OC and EVOO-PPRF

**Figure 5:**
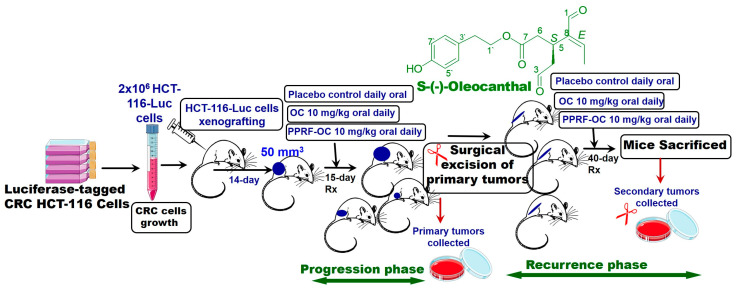
The study design of OC and PPRF in vivo assessments against the CRC HCT-116-Luc progression and recurrence in male nude mice.

Fifteen athymic nude mice were used to create a xenograft model to assess the anti-CRC activity of OC either in pure- or crude-EVOO-derived PPRF form, versus the placebo control (*n* = 5 per group). The study included the CRC progression assessment (15 days’ dosing), after which the animals were subjected to primary-tumor surgical excisions, and the same animals were used, continuing the same dosing regimen, to assess the recurrence suppressive activity (Figure 5). Two million HCT-116-Luc cells were xenografted subcutaneously into each nude mouse, into the back-right flank. When the tumors reached 50 mm^3^, oral treatments started and continued for 15 days (progression phase). The mice were then subjected to primary-tumor surgical excisions, and dosing continued for 40 additional days to assess the recurrence suppressive activity (Figure 5, recurrence phase).

### 3.8. Effect of OC and EVOO-PPRF on HCT-116-Luc CRC Cels Progression in a Nude Mouse Xenograft Model

**Figure 6:**
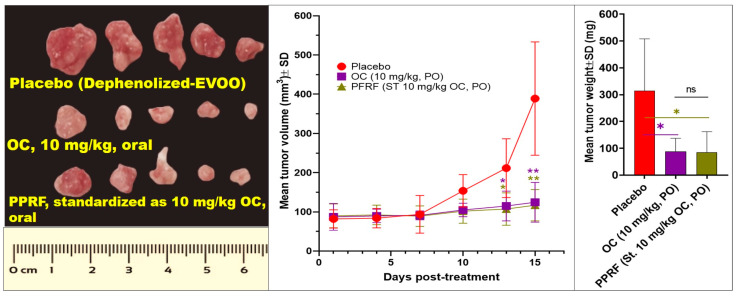
Daily 10 mg/kg oral OC for 15 days reduced the CRC HCT-116-Luc progression in male nude mice. Statistical significance was assessed using a paired *t*-test: ** *p* < 0.01, * *p* < 0.05, and ns indicates no significance.

Daily oral OC 10 mg/kg and PPRF, with OC content equivalent to 10 mg/kg, was used for 15 days, and effectively suppressed HCT-116-Luc tumor weight by 72.5% and 73.5%, respectively, in male nude mice versus the placebo control (EVOO without phenolics extracted out, dephenolized EVOO, Figure 6). OC and PPRF treatments inhibited 68% and 71.1%, respectively, of HCT-116-Luc tumor volume, versus the placebo control treated group (Figure 6).

### 3.9. OC and PPRF Effectively Suppressed HCT-116-Luc CRC Recurrences

**Figure 7:**
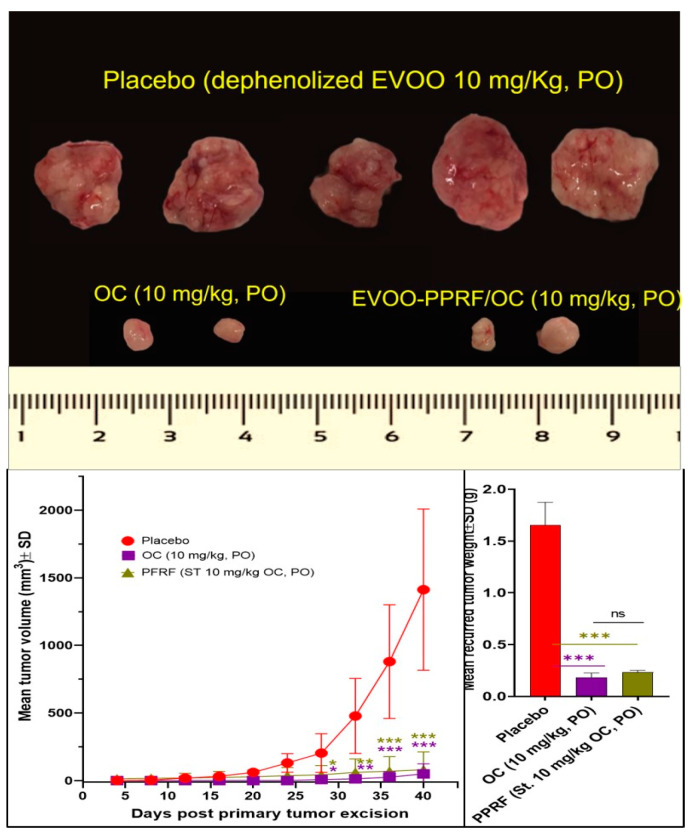
The suppressive effects of OC on CRC recurrences. Continued daily 10 mg/kg oral OC and PPRF treatments after primary-tumor surgical excision for 40 days reduced HCT-116-Luc cell locoregional-tumor recurrences in nude mice. Statistical significance was assessed using a paired *t*-test: *** *p* < 0.001, ** *p* < 0.01, * *p* < 0.05, and ns indicates no significance.

**Figure 8:**
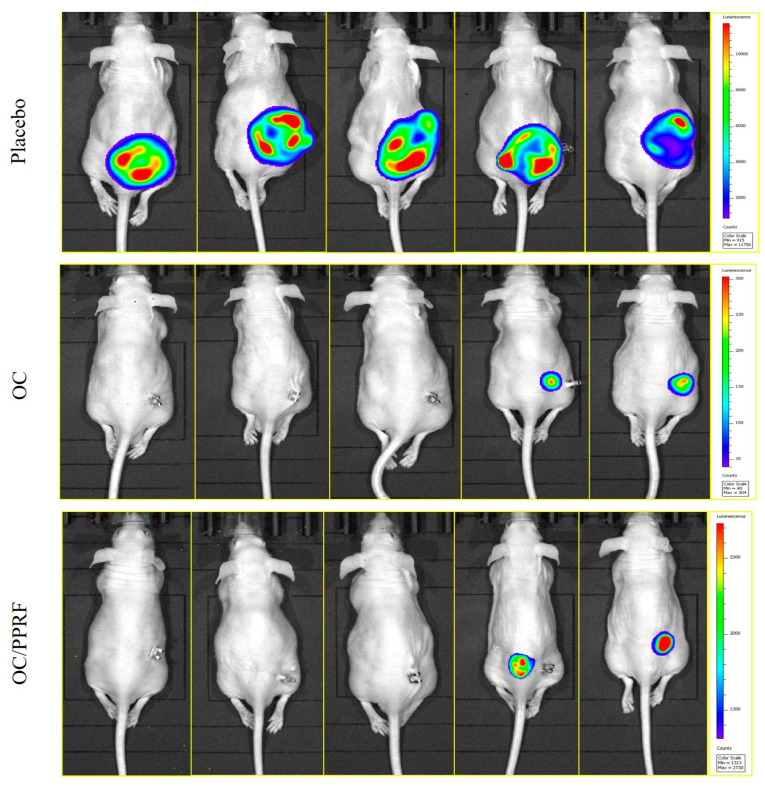
Animal imaging before sacrifice, after the recurrence study conclusion, showing 5/5 locoregional recurrence in placebo control versus 2/5 in OC and PPRF treatments.

Animals used for the tumor progression model were subjected to primary-tumor surgical excision, and the animals continued with daily oral 10 mg/kg OC, PPRF, and placebo treatments until the mice were sacrificed 40 days after the surgery. OC and PPRF treatments reduced the HCT-116-Luc locoregional recurring-tumor weight by 88.6% and 86.1%, respectively, versus the placebo control-treated tumors (Figure 7). Meanwhile, each of the OC and PPRF treatments reduced 87% of the HCT-116-Luc locoregional recurring-tumor volume versus the placebo control-treated tumors (Figure 7). The placebo control-treated mice group showed five out of five locoregional tumor recurrence at the study end, versus only two out of five in the OC- and PPRF-treated mice groups (Figure 8). Locoregional recurrence tumors started initiation in the placebo-treated group during days 12–28, after the primary-tumor excision surgery (Figure 7). The first locoregional recurrence tumors in both OC- and PPRF-treated mouse groups started on day 28, indicating a 16-day tumor-recurrence latency compared to the placebo control-treated group (Figure 7). Morphological examination of the collected primary and recurrence tumors revealed notably reduced vascularity in OC- and PPRF- treated groups, compared to the placebo control tumors (Figure 6 and Figure 7).

All OC and PPRF-treated mice survived over the full-experiment days, 40 days after primary-tumor surgical-excision surgery (Figure 8). Meanwhile, three out of five mice in the placebo control group reached the tumor volume cut-off limit, and had to be sacrificed on days 25, 33, and 37 after the tumor-excision surgical day (Supplementary Figure S3). Minute distant recurrences in lung, brain and spleen were observed only in the placebo-treated mice group during organ imaging collected after animal sacrifice (Supplementary Figure S4). Unlike the placebo-control mice group, all OC- and PPRF-treated mice did not show any bioluminescent distant-tumor recurrences on any organ (Supplementary Figure S4).

### 3.10. OC and PPRF Effectively Suppressed the Epression Levels of SMYD2, EZH2 and c-MET in Collected HCT-116 Cell Primary Tumors

**Figure 9:**
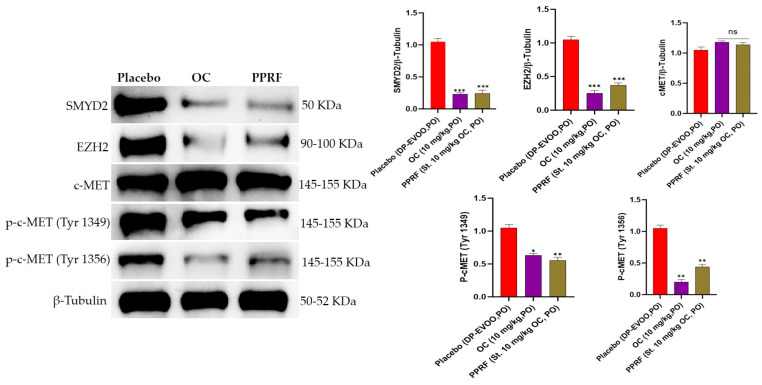
Molecular effects of OC and PPRF on CRC HCT-116 primary tumors. Western blotting experiments indicated that OC and PPRF treatments notably suppressed the expression levels of SMYD2, EZH2, and activated c-MET in collected HCT-116-Luc primary tumors. Bar graphs depict normalized protein levels as mean ± SEM. Statistical significance was assessed using a paired *t*-test: *** *p* < 0.001, ** *p* < 0.01, * *p* < 0.05, and ns indicates no significance.

Western blotting analyses of collected animal primary tumors collected by surgical excision indicated significant suppression of the expression level of SMYD2, EZH2, and p-c-MET (Tyrosines 1349 and 1356, Figure 9). OC and PPRF treatments reduced the total SMYD2 expression level in HCT-116 tumors by 79% and 77.4%, respectively. OC and PPRF treatments reduced the total EZH2 expression level in CRC tumors by 77.3% and 66.7%, respectively. OC and PPRF treatments did not significantly affect the total c-MET expression level, but reduced the p-c-MET tyrosine-1349 expression level in HCT-116 tumors by 40.6% and 46.9%, respectively. OC and PPRF treatments significantly reduced the p-c-MET tyrosine-1349 expression level in HCT-116 tumors by 80.7% and 58.1%, respectively. These results are consistent with the in vitro data showing a slight preference for suppressive effects of PPRF over pure OC on p-c-MET tyrosine-1349 and a preference for PPRF over pure OC on p-c-MET tyrosine-1356 (Figure 9). 

### 3.11. Effect of OC and PPRF Treatments on Mean Animal Body Weight Throughout Progression and Recurrence Phases of the Study Design

Daily oral treatments for mice with OC and PPRF over the study course, 54 days, did not result in any significant changes in animal mean body weight, compared to the placebo group (Supplementary Figure S5), indicating that OC and PPRF, at their effective therapeutic doses, did not induce notable adverse effects on general animal health or metabolic function. This lack of animal body-weight alterations between the study groups serves as a positive indicator of OC and PPRF safety profiles.

## 4. Discussion

### 4.1. Significance and Study Rationale 

CRC is the third topmost US abundant malignancy in both genders, with a high recurrence rate and progressive resistance to existing therapies. The *KRAS* mutations were detected in most CRCs, inducing worse prognosis and high resistance to EGFR-targeted therapies [16,17,18]. Current therapies do not effectively eradicate the disease and, therefore, novel interventions are direly needed. Epidemiological studies indicate the low CRC and other cancers’ incidence in Mediterranean populations, which was attributed to the frequent intake of the Mediterranean diet rich in EVOO [30,33]. OC is a monophenolic bioactive secoiridoid naturally occurring in EVOO. OC had reported anticancer activities against several malignancies, including in vitro activities against some CRC cell lines [31,32,33]. Despite the promise of epidemiological data that show the potential of EVOO phenolics for the control of CRC in Mediterranean populations, no studies so far have assessed the in vivo anti-CRC efficacy of OC, the most abundant, potent and bioactive EVOO phenolic.

### 4.2. Uniqueness of the OC Anti-CRC Molecular Targets 

This study team validated the c-MET receptor tyrosine kinase (RTK) and the lysine methyl transferase SMYD2 as unique molecular targets for OC in breast and prostate cancers, respectively [27,28,29]. c-MET is usually overexpressed in metastatic CRC clinical samples, which correlated with poor patient prognosis and a high invasive profile [18,19,20]. Thus, c-MET is a valid molecular target for CRC control, yet no c-MET inhibitors have been approved for anti-CRC clinical use so far. Crizotinib (CRZ), an FDA-approved dual c-MET/ALK inhibitor, induced PUMA and Bim, along with p53 stabilization and DNA damage in p53 wild-type and EpCAM-overexpressing CRC [36,37]. CRZ, combined with regorafenib/PD-1 inhibitor, proved active against metastatic *BRAF V600*EMT CRC, with amplified c-MET and TPM4-ALK fusion [38].

The lysine methyltransferase SMYD2 methylates numerous histone and non-histone protein substrates, including the trimethylation of the master transcriptional regulator EZH2 at K307, promoting aggressive tumorigenesis [12,39]. The methyl-lysine-containing proteins are recognized by methyllysine readers, activating numerous oncogenic downstream cascades [10]. Aberrant activation of SMYD2 suppressed the adenomatous polyposis coli 2 (APC2), which activated the Wnt/β-catenin pathway, and induced epithelial–mesenchymal transition in CRC [40]. Mechanistically, low APC2 expression in CRC cells was attributed to SMYD2-mediated DNA methylation modification [41]. Clinically, SMYD2 was aberrantly dysregulated in patients with invasive and metastatic CRC [21,22]. Thus, dually targeting the c-MET RTK and the SMYD2-EZH2 axis represents a novel strategy to control CRC.

### 4.3. The Recognition of OC as the Most Active Olive Phenolic Anti-CRC Hit, a Potency Comparison with Established CRC Therapies, and an Overview of Its Safety and Selectivity 

Screening of the expression levels of diverse CRC cell lines defined the KRAS*^G13D^-* PIK3CA*^H1047R^* mutated HCT-116 cells [34] with nearly 1.5-fold and 5-fold SMYD2 and c-MET expression levels, respectively, compared to the non-tumorigenic human colon epithelial CCD 841 CoN cells (Figure 1). A library of seven olive phenolics, including multiple secoiridoids and lignans were screened in vitro against four diverse CRC cell lines. This screening identified OC as the topmost antiproliferative active olive phenolic against CRC cell lines. Further focused assessment of OC and PPRF with high OC content supported the anti-CRC superiority of OC over the rest of the olive phenolics, either in pure form or in crude PPRF form (Figure 2 and Figure 3). The in vitro OC anti-CRC activity was compared with a wide range of targeted therapies, including the first- and third-generation EGFR inhibitors gefitinib and osimertinib, respectively, and the experimental standard SMYD2 and c-MET inhibitors BAY-598 and SU11274, respectively (Table 1). OC anti-CRC activity was compared with standard chemotherapeutics, including the taxane paclitaxel and the anthracycline antibiotic doxorubicin, in addition to 5-fluorouracil (Table 1). Paclitaxel and doxorubicin showed superior potency against the four tested CRC cell lines. OC was nearly comparable to the anti-CRC potency of 5-fluorouracil, osimertinib, and SU11274 (Table 1). OC showed better in vitro anti-CRC activity than the first-generation EGFR antagonist gefitinib and the SMYD2 inhibitor BAY-598. OC proved highly selective to CRC cells, as compared to effects on the viability of the non-tumorigenic human colon epithelial CCD 841 CoN cells, with an IC_50_ value 7.5-fold higher than its best cytotoxic IC_50_ value against CRC. Monitoring the animals’ body-weight average over the study course showed no suppressive treatment effects (Supplementary Figure S5), suggesting a potential preliminary safety profile. This clearly highlighted the translation potential of OC for use in controlling CRC, with superior safety and low off-target tendency, based on historical human food consumption of EVOO, in vitro data, a lack of treated animals’ body-weight change over the study course (Supplementary Figure S5), and published in vivo acute-safety studies [42]. OC and EVOO PPRF effectively inhibited HCT-116 migration (Supplementary Figure S1). OC and EVOO PPRF also potently suppressed HCT-116 colony formation (Supplementary Figure S2). This assay closely mimics the in vivo clonogenicity of the dispersed circulating tumor cells at a distant site. These tumor cells likely escaped the surgery, penetrated the tumor–animal tissue microenvironment and/or resisted the therapy, causing the distant recurrences [43]. The in vitro migration and clonogenicity-suppressing effects of OC and EVOO PPRF bode well with their in vivo distant-recurrence suppressive activities.

OC and PPRF inhibited the in vitro expression of SMYD2 and EZH2 in HCT-116 cells at subtoxic IC_50_ treatment ranges (Figure 4). Pure OC was much more potent than EVOO PPRF in suppressing the expression levels of SMYD2 and EZH2. Although both proteins are lysine methyltransferases, SMYD2 directly methylates EZH2 at K307, promoting tumorigenesis [12,39]. Dually suppressing both SMYD2 and EZH2 is translationally advantageous to prevent a wide array of downstream substrate protein activation.

c-MET is the only receptor tyrosine kinase with a MET binding domain (MBD), which engages in diverse protein–protein interactions [44,45,46,47,48]. The MBD tyrosines 1349 and 1356 offer a proper docking platform to recruit important signaling proteins like Src homology-2 domain, phosphotyrosine-binding domain, and other critical MBD adapter proteins required for motility activation, including GAB1, GRB2, phospholipase C, and SRC [44,45,46,47]. The c-MET kinase domain activation also initiates several downstream pathways, including MAPK, PI3K/Akt, STAT, and NFκB, inducing cell proliferation, survival, EMT, scattering, invasion, protection from apoptosis, and angiogenesis. Inhibition of MBD tyrosine 1349 mostly affects the motility direction, unlike the inhibition of MBD tyrosine 1356, which mainly interferes with the tumor growth and survival pathways direction [46,48]. OC effectively suppressed the MBD p-c-MET tyrosine 1356 more than PPRF, which suggests a preference for tumor growth/survival inhibition over the use of crude PPRF. The EVOO PPRF was more effective than pure OC in inhibiting the p-c-MET tyrosine 1349 in HCT-116 cells and collected primary tumors (Figure 4 and Figure 9), justifying its preference for the tumor-motility suppressive effects. This effect might be attributed to the possible existence of minor EVOO ingredient(s) selective for MBD tyrosine-1349 inhibition, offering preference for the crude EVOO-derived PPRF over pure OC in distant-CRC-recurrence prevention (Figure 4 and Figure 9). This result highlights the potential of the high-phenolic content EVOO to control CRC motility. The fact that OC and PPRF potently suppressed the expression of SMYD2, EZH2, and c-MET tyrosine 1349 and 1356 in collected primary tumors (Figure 9), validated their promotion to the lead rank, and proved the pharmacodynamic effects, as well as provided the pharmacological justification and evidence for their molecular targets in suppressing CRC progression, recurrences, and motility.

### 4.4. Hit-to-Lead Validation of OC as a Novel Anti-CRC Entity 

The most sensitive CRC cell line, HCT-116, was selected for the in vivo xenograft model, to assess the anti-CRC progression and recurrence of both pure OC, and crude EVOO-derived PPRF versus the placebo EVOO, from which all phenolics are extracted out (Figure 6, Figure 7, Figure 8 and Figure 9). Both OC and PPRF were nearly similar in significantly suppressing more than 70% of HCT-116 primary tumor progression, validating OC and PPRF as potential anti-CRC lead nutraceuticals. Similarly, both OC and PPRF showed effective (>85% of tumor weight and volume) locoregional-recurrence inhibition, allowing recurrence only in two out of five mice in each group, unlike the placebo-treated group, which developed massive locoregional recurrence tumors in five out of five of the group mice. The first locoregional recurrence tumors in both OC- and PPRF-treated mouse groups were delayed for 16 days after tumor recurrence started in the placebo group. It is well-established that each 2.6 days of a mouse life is equivalent to one human year [49]. This indicates that the tumor-recurrence latency extension in OC- and PPRF-treated groups, compared to the placebo control-treated group, is equivalent to 6.2 years of human life [49]. Collected primary and recurrence tumors notably showed morphologically reduced vascularity in OC- and PPRF-treated groups, compared to the placebo control, suggesting potent inhibition of the angiogenic tumor ability (Figure 8 and Figure 9). All OC- and PPRF-treated mice did not show any distant tumor recurrences in any organ, unlike the placebo group, which showed micrometastatic tumor foci in brain, lung and spleen (Supplementary Figure S4). This result strongly validates the potent CRC locoregional- and distant-recurrence suppressive activity of OC, whether in pure form or crude as EVOO-derived PPRF, highlighting their potential for the CRC control.

### 4.5. OC Versus EVOO: Anti-CRC Drug Entity, Food or Nutraceutical? 

Results definitely showed the equipotency for the pure- and crude-OC form tested as PPRF extracted from OC-rich EVOO. Mimicking the used therapeutic dose in this study, 10 mg/kg, for use in humans with an average 70 kg body weight and assuming the use of a high-phenolic content quality EVOO brand, with nearly 1 g OC/L content, the needed daily anti-CRC therapeutic EVOO volume is translated to 700 mL, which is not a realistic volume for the regular food-intake use. There is definitely an urgent need for future studies to determine whether OC lower doses will maintain the same 10 mg/kg dosing potency in vivo. Lower OC dosing should be tested in future, both in therapeutic and preventive modes, to determine if the consumption of regular EVOO food amounts can prevent and/or eradicate CRC. This study provided evidence for the dire need for pure-OC and/or crude-EVOO-derived OC-rich PPRF nutraceuticals for intended use by the CRC patients and survivors, to control their disease and prevent relapse.

## 5. Conclusions

This study validated the pure OC and crude EVOO-derived PPFR form as potential leads for the control of CRC progression and recurrences. The study results correlated well with the epidemiological studies that suggested the lower CRC incidence in the Mediterranean populations, due to their progressive intake of EVOO phenolics in their diets. The results open preclinical directions for the development and use of OC and PPRF as CRC nutraceutical lead interventions. OC and EVOO phenolics targeted critically important molecular targets, including the lysine methyltransferases SMYD2 and EZH2, which activate several downstream protein substrates important for CRC growth, progression and motility. c-MET is the only RTK that possess a protein–protein binding hub, MBD, intended to recruit and activate several downstream mitogenic pathways. OC and PPRF effectively inhibited the autophosphorylation of the MBD tyrosines 1349 and 1356, preventing their subsequent activation of critical downstream pathways in progression and motility directions. The combined inhibition of the lysine methyltransferases SMYD2-EZH2 and the RTK c-MET is a novel molecular mechanism, making the EVOO phenolics, represented by OC, exceptionally unique anti-CRC nutraceuticals, useful for both CRC patients and survivors.

## Figures and Tables

**Table 1:** Comparison of the OC in vitro antiproliferative activities versus anticancer drugs *.

Tested Compound	CRC Cell Line, IC_50_ (µM)			
	HCT-116	SW48	COLO-320DM	WiDr
PTX	0.005	0.007	0.01	0.10
DOX	0.17	0.19	0.21	0.4
OSI	3.1	1.25	5.7	7.0
5-FU	3.0	3.6	4.1	5.0
OC	4.2	4.9	9.8	14.5
BAY-598	8.0	13.13	NT	9.18
SU11274	4.9	7.9	8.7	13.2
GFT	5.8	8.0	11.1	12.4

* PTX (Paclitaxel), DOX (Doxorubicin HCl), OSI (Osimertinib), 5-FU (5-Fluorouracil), BAY-598 (Investigational SMYD2 inhibitor), SU11274 (Investigational c-MET inhibitor), GFT (Gefitinib).

## Data Availability

All data used to support the findings of this study made available in this publication as figures, tables, or Supplementary Materials.

## Supplementary Materials

The following supporting information can be downloaded at: https://www.mdpi.com/article/10.3390/nu17030397/s1, Figures S1: Effects of OC and PPRF on the migration of the CRC HCT-116 cells using wound-healing assay; Figure S2: Effects of PPRF and OC on the clonogenicity of HCT-116 cells using colony formation assay; Figure S3: Kaplan-Meier survival plot comparing the percent mice survival at the study end for OC and PPRF treatments versus placebo control; Figure S4: Collected animal organs showing distant recurrences in 3 out 5 placebo control (lung-brain-spleen) versus 0 out of 5 in OC and PPRF-treated mouse groups; Figure S5: Overview monitoring of the effects of OC and PPRF treatments on mice body weight throughout the progression and recurrence phases of the study; Figure S6: SMYD2 and c-MET total level expressions in CRC cells HCT 116, COLO 320 DM, SW48, WiDr, and non-tumorigenic colon epithelial cells CCD 841 CoN cells raw Western blot images; Figure S7: OC and PPRF effects on expression levels of SMYD2, EZH2, total, and activated c-MET in HCT-116 cells raw Western blot images; Figure S8: OC and PPRF effects on expression levels of SMYD2, EZH2, total, and activated c-MET (tyrosines-1349 and 1356) in collected HCT-116 CRC cells primary tumors raw Western blot images.
