# Supplementary material for: The Olive Oil Phenolic S-(-)-Oleocanthal Suppresses Colorectal Cancer Progression and Recurrence by Modulating SMYD2-EZH2 and c-MET Activation
Source: Nutrients. 2025 Jan 22;17(3):397. doi: 10.3390/nu17030397 (PMC11821053; doi:10.3390/nu17030397)
Supplement: Supplementary file 1 [file nutrients-17-00397-s001.zip › nutrients-3398931-supplementary.pdf]

nutrients

MDPI

## Supplementary Materials

# The Olive Oil Phenolic *S*-(-)-Oleocanthal Suppresses Colorectal Cancer Progression and Recurrence by Modulating SMYD2-EZH2 and c-MET Activation

**Md Towhidul Islam Tarun, Heba E. Elsayed, Hassan Y. Ebrahim and Khalid A. El Sayed ***

School of Basic Pharmaceutical and Toxicological Sciences, College of Pharmacy, University of Louisiana at Monroe, 1800 Bienville Drive, Monroe, LA 71201, USA; tarunmt@warhawks.ulm.edu (M.T.T.); hebassan_2005@yahoo.com (H.E.); hebrahim@ulm.vcom.edu (H.Y.E.)

* Correspondence: elsayed@ulm.edu; Tel.: +1-318-342-1725

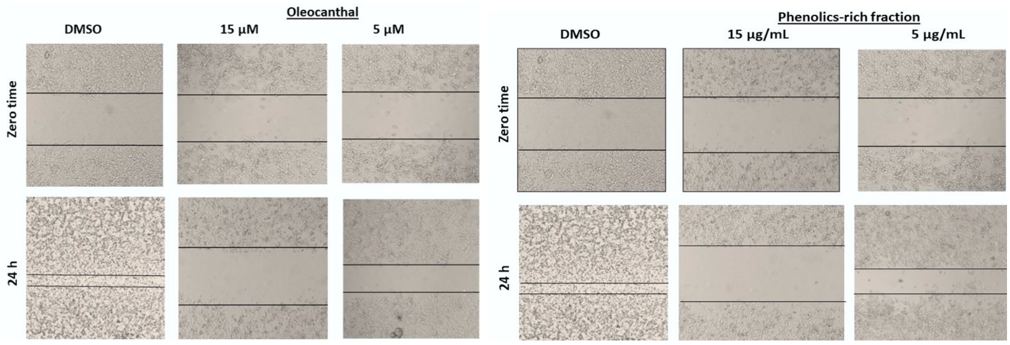


**Figure S1.** Effects of OC and PPRF on the migration of the CRC HCT-116 cells using wound-healing assay.

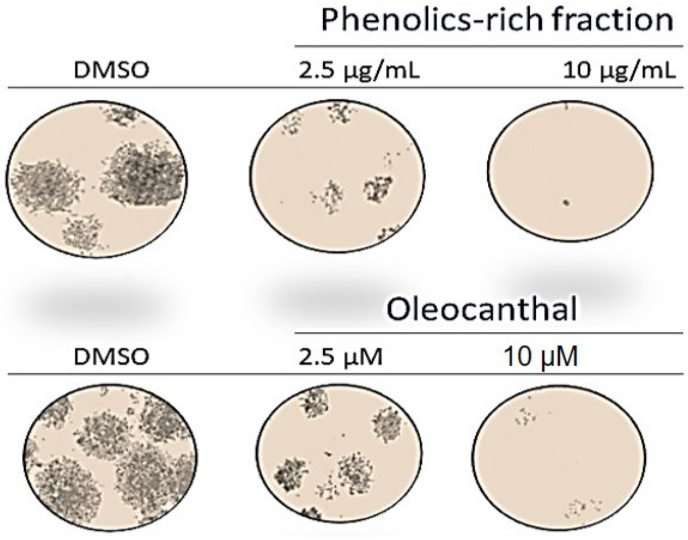


**Figure S2.** Effects of PPRF and OC on the clonogenicity of HCT-116 cells using colony formation assay.

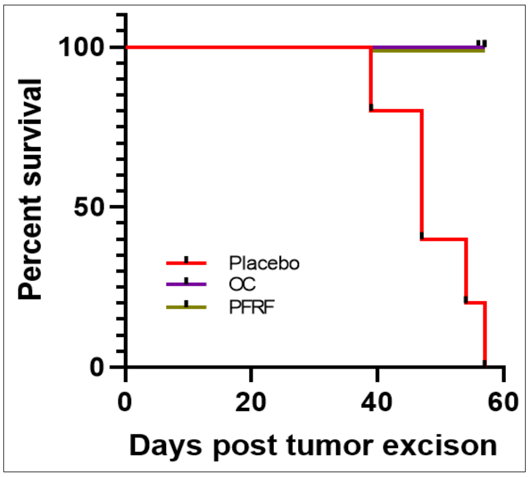


**Figure S3.** Kaplan-Meier survival plot comparing the percent mice survival at the study end for OC and PPRF treatments versus placebo control.

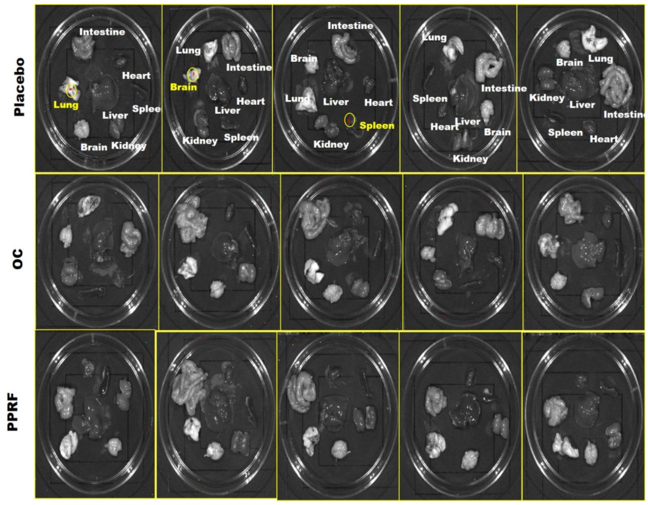


**Figure S4.** Collected animal organs showing distant recurrences in 3 out 5 placebo control (lung-brain-spleen) versus 0 out of 5 in OC and PPRF-treated mouse groups.

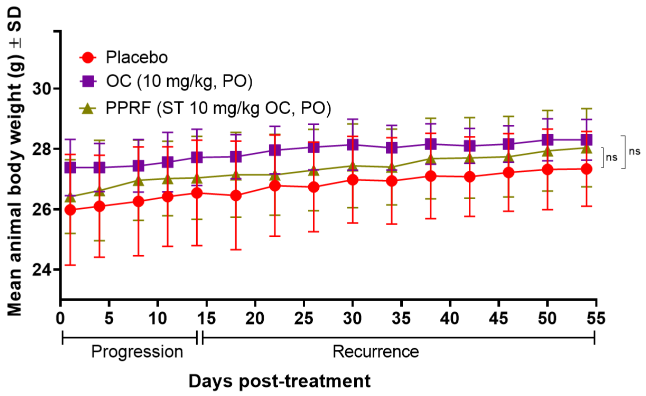


**Figure S5.** Overview monitoring of the effects of OC and PPRF treatments on mice body weight throughout the progression and recurrence phases of the study. Data are presented as mean ± SD. "ns" indicates statistical non-significance at $p < 0.05$.

**SMYD2**

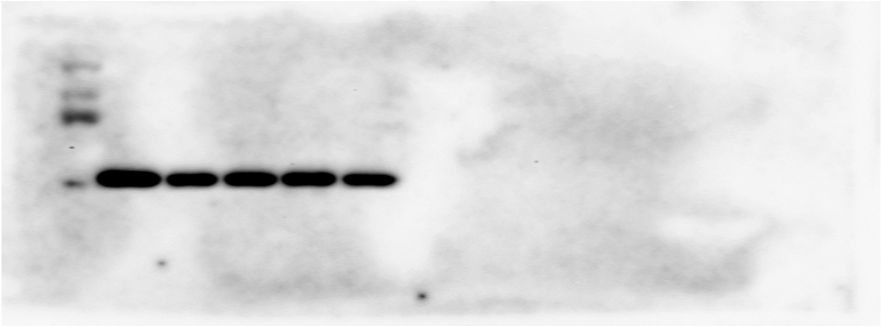

**c-MET**

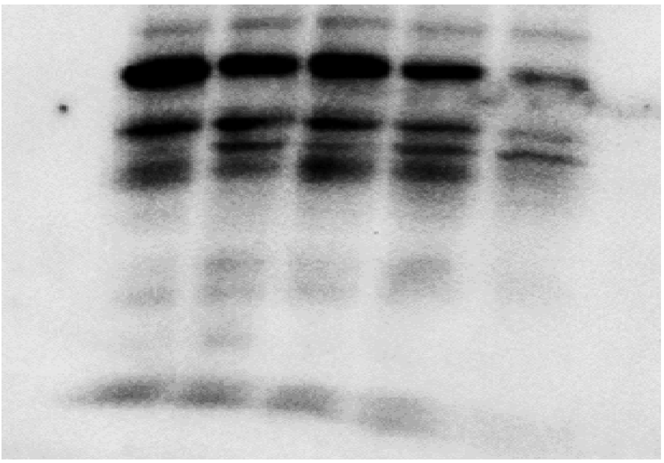

**β-Tubulin**

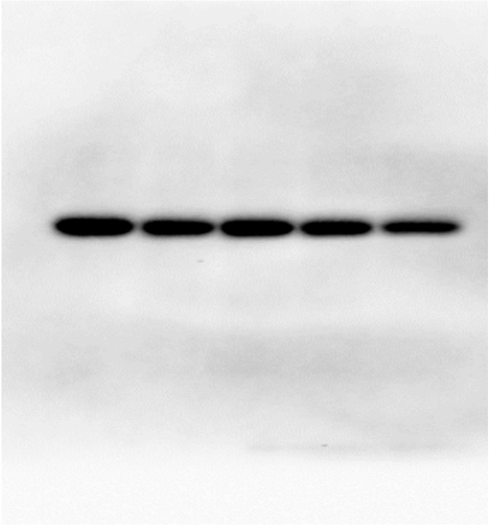

**Figure S6.** SMYD2 and c-MET total level expressions in CRC cells HCT 116, COLO 320 DM, SW48, WiDr, and non-tumorigenic colon epithelial cells CCD 841 CoN cells raw Western blot images.

**<u>OC</u>**

**SMYD2**

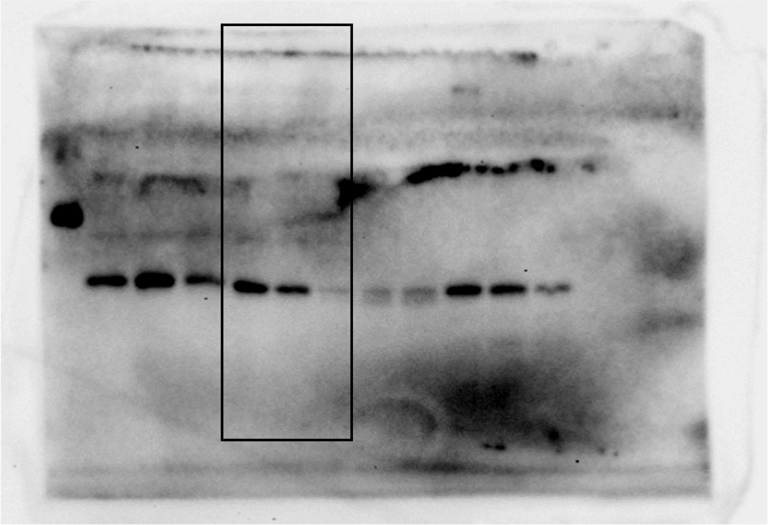

**EZH2**

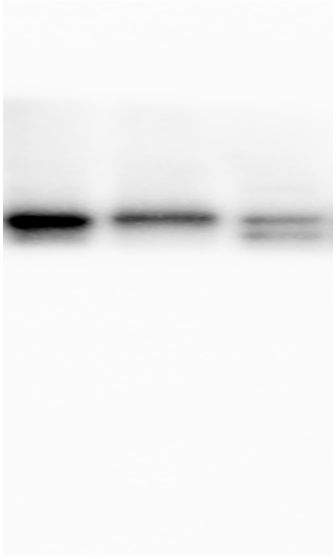

**c-MET**

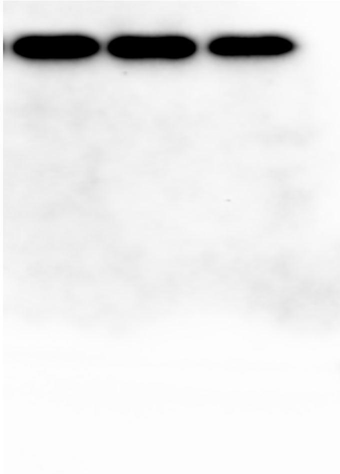

**p-MET (1349)**

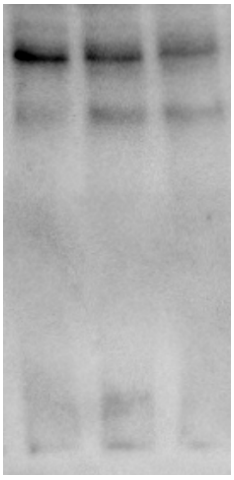

**p-MET (1356)**

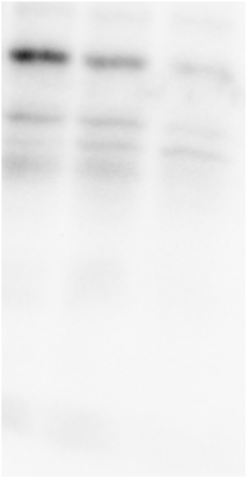

**PPRF**

**SMYD2**

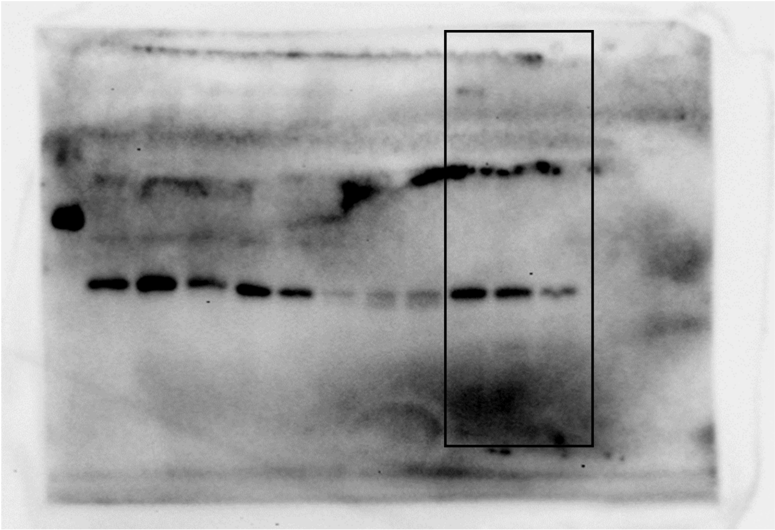

**EZH2**

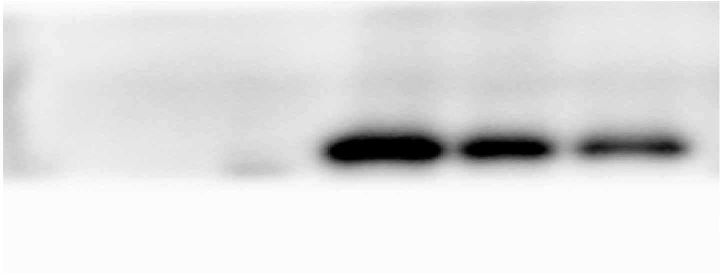

**c-MET**

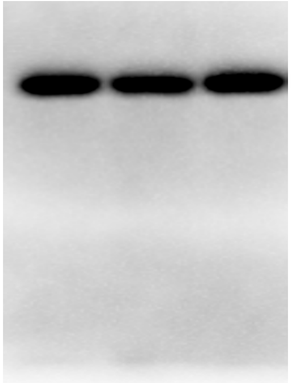

**p-MET (1349)**

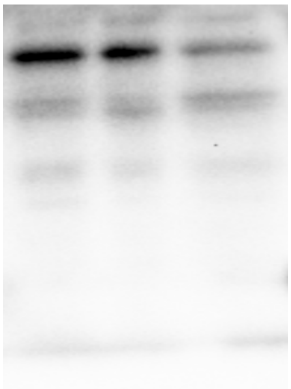

**p-MET (1356)**

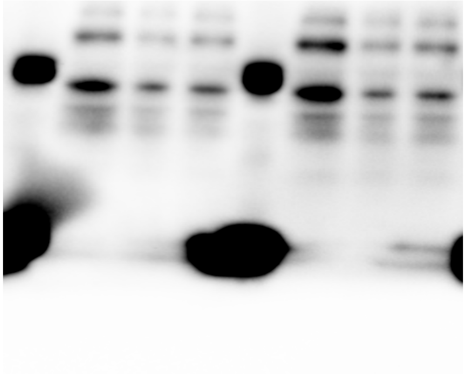

**β-Tubulin**

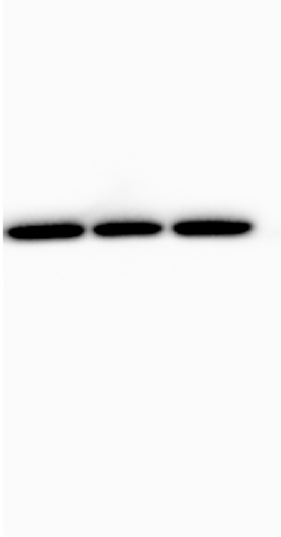

**Figure S7.** OC and PPRF effects on expression levels of SMYD2, EZH2, total, and activated c-MET in HCT-116 cells raw Western blot images.

<u>**OC**</u>

**SMYD2**

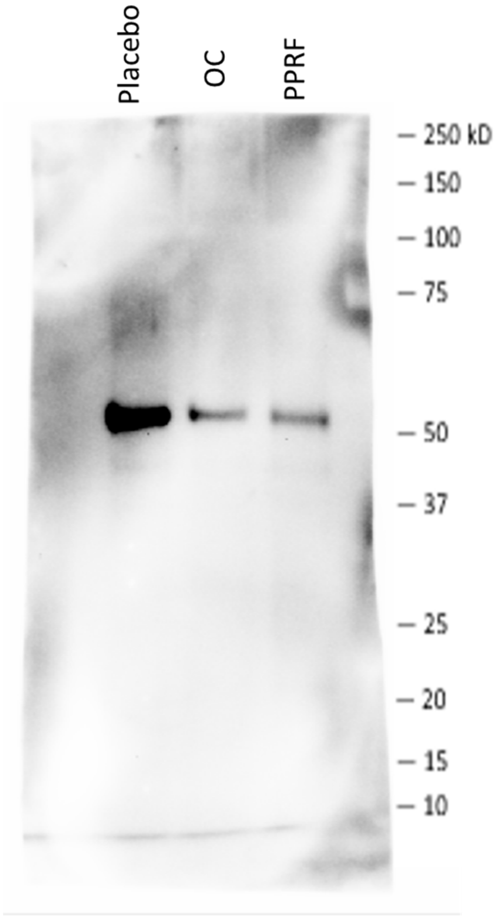


**EZH2**

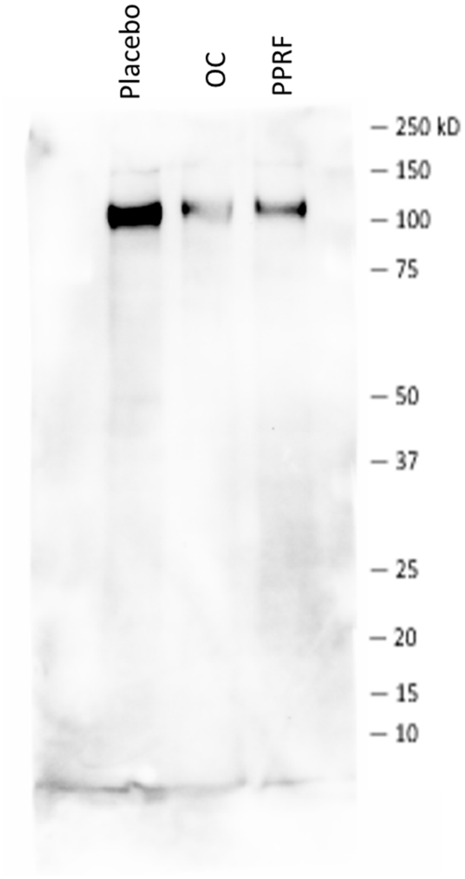

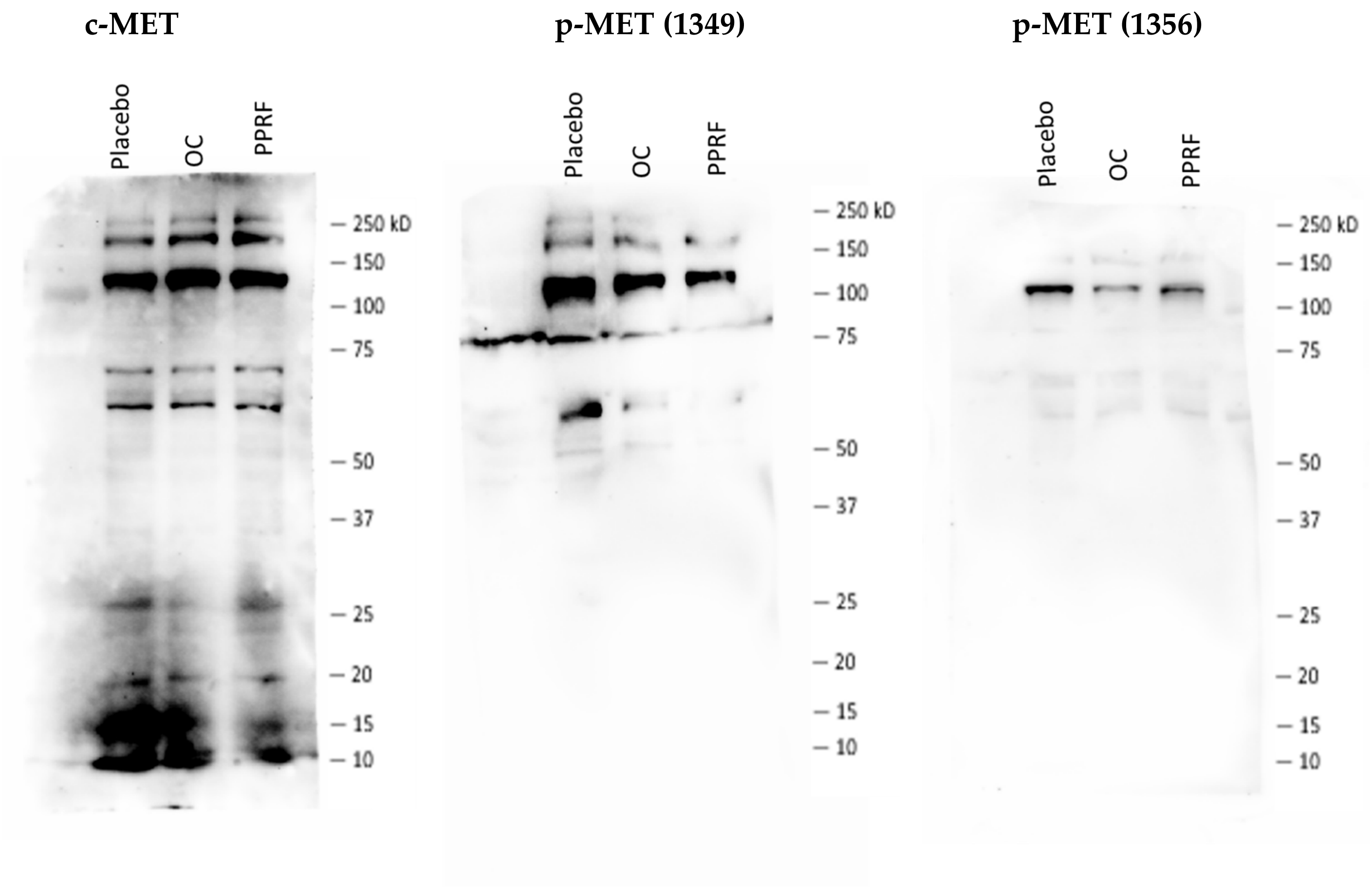
c-MET
p-MET (1349)
p-MET (1356)
Placebo
OC
PPRF
250 kD
150
100
75
50
37
25
20
15
10

**β-Tubulin**

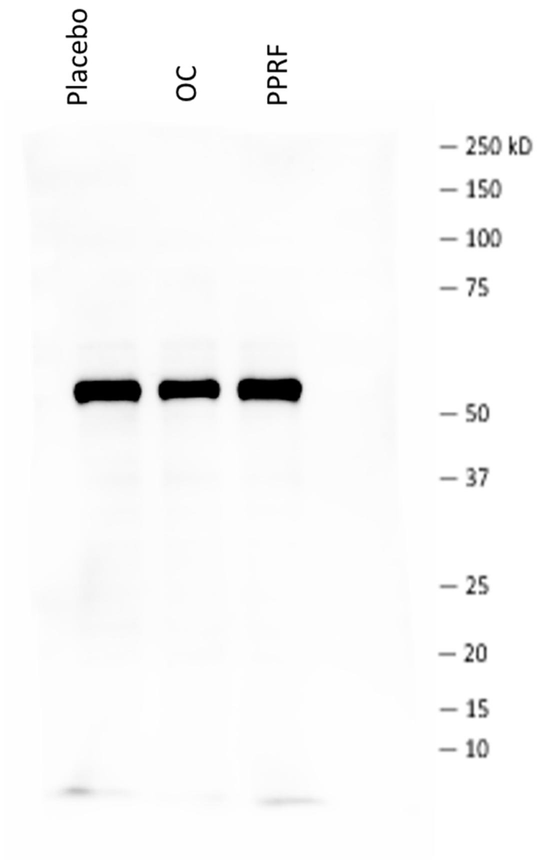


**Figure S8.** OC and PPRF effects on expression levels of SMYD2, EZH2, total, and activated c-MET (tyrosines-1349 and 1356) in collected HCT-116 CRC cells primary tumors raw Western blot images.
